# Effectiveness of Adding GLP‐1 Receptor Agonists to SGLT2 Inhibitor Therapy in Chronic Kidney Disease: A Population Based Study

**DOI:** 10.1111/dom.71375

**Published:** 2026-09-27

**Authors:** Hussain Salim, Shaheer Qureshi, Gaurav S. Gulsin, Habib R. Khan, Saad Ahmed Waqas, Gerry P. McCann, Amit Garg, Mohammed Shurrab, Kamlesh Khunti, Muhammad Rashid

**Affiliations:** ^1^ Department of Medicine Dow University of Health Sciences Karachi Pakistan; ^2^ National Institute of Health and Care Research, Leicester Biomedical Research Centre Glenfield Hospital Leicester UK; ^3^ Department of Cardiovascular Sciences, British Heart Foundation Cardiovascular Research Centre University of Leicester Leicester UK; ^4^ Division of Cardiology, Department of Medicine, Schulich School of Medicine and Dentistry Western University London Ontario Canada; ^5^ Division of Nephrology, Department of Medicine, Schulich School of Medicine and Dentistry Western University London Ontario Canada; ^6^ Cardiology Department Health Sciences North, Northern Ontario School of Medicine University Sudbury Ontario Canada; ^7^ Diabetes Research Centre University of Leicester Leicester UK; ^8^ Keele Cardiovascular Research Group, Centre for Prognosis Research, School of Medicine Keele University Keele UK

**Keywords:** dapagliflozin, empagliflozin, glucagon antagonist, semaglutide, SGLT2 inhibitor

## Abstract

**Background:**

Individually, sodium–glucose co‐transporter‐2 inhibitors (SGLT2i) and glucagon‐like peptide‐1 receptor agonists (GLP‐1RAs) have been shown to reduce major kidney and cardiovascular events in patients with chronic kidney disease (CKD). However, evidence supporting combined GLP‐1RA and SGLT2i therapy in this population remains limited.

**Methods:**

We conducted a retrospective cohort study using the TriNetX Global Collaborative Network, including adults with CKD treated with SGLT2i alone or SGLT2i with subsequent initiation of GLP‐1RA between June 2020 and December 2023. Propensity score matching (1:1) adjusted for demographics, comorbidities, medications, and laboratory values. The primary outcome was major adverse kidney events (MAKE); secondary outcomes included all‐cause mortality and major adverse cardiovascular events (MACE). Time‐to‐event analyses were performed using Cox proportional hazards models.

**Results:**

Among 112 596 patients with CKD, 16460 received SGLT2i and GLP‐1RA combination therapy, and 96 136 received SGLT2i monotherapy. The matched cohort included 32 448 patients (16 224 per group). Over a median follow‐up of 12 months, the addition of a GLP‐1RA was associated with lower rates of the primary outcome of MAKE (HR 0.70; 95% CI 0.65–0.74; *p* < 0.001) compared with SGLT2i monotherapy. Combination therapy was also associated with lower risks of MACE (HR 0.83; 95% CI 0.78–0.87) and all‐cause mortality (HR 0.55; 95% CI 0.50–0.61). Benefits were consistent across CKD stages and diabetes status, with significantly more pronounced effects observed in patients with obesity, heart failure, and ischemic heart disease. Combination therapy was associated with increased gastrointestinal symptoms, genital infections, and retinopathy progression, but lower risks of volume‐depletion events and acute kidney injury.

**Conclusions:**

In this real‐world CKD cohort, GLP‐1RA added to SGLT2i therapy was associated with substantial incremental reductions in kidney disease progression and cardiovascular events. These findings suggest an additive cardiorenal benefit, particularly in patients with obesity, supporting the evaluation of combination therapy in future randomised trials.

## 1 Introduction

Chronic kidney disease (CKD) is a major global health problem affecting over 10% of the world's population and has evolved from a complication of diabetes to a primary target for cardiometabolic interventions [1]. While sodium‐glucose co‐transporter‐2 inhibitors (SGLT2i) have transformed the standard of care, demonstrating robust reductions in progression to end‐stage kidney failure and mortality in randomised controlled trials, they do not eliminate the risk of progression [2]. A significant proportion of patients continue to experience declining kidney function and adverse cardiovascular events, highlighting an unmet need for adjunctive therapies that address residual risk [3, 4, 5, 6, 7].

More recently, glucagon‐like peptide‐1 receptor agonists (GLP‐1RAs) have also emerged as effective renoprotective agents. Cardiovascular outcomes trials, including LEADER, SUSTAIN‐6, REWIND, and AMPLITUDE‐O, initially signalled kidney benefits of GLP‐1RAs, a finding recently solidified by the dedicated kidney outcome trial, FLOW (Effects of Semaglutide on CKD in Patients with Type 2 Diabetes), which was stopped early for efficacy reasons [8, 9, 10, 11, 12]. The biologic rationale for combining these two classes is compelling. Effects of GLP‐1RA and SGLT2i were assessed on top of standard ACEi/ARB therapy. SGLT2 inhibitors lower intraglomerular pressure, while GLP‐1RAs add anti‐inflammatory and natriuretic actions [13, 14]. This distinct mechanistic divergence suggests that dual therapy could yield mechanistically complementary protection against CKD progression [15].

Despite this mechanistic promise and the increasing concurrent use of these agents in routine practice, clinical evidence supporting combination therapy remains sparse. Current guidelines from the American Diabetes Association (ADA) and Kidney Disease: Improving Global Outcomes (KDIGO) endorse both classes individually but lack specific recommendations for dual treatment. This is largely because major SGLT2i trials included very few participants on GLP‐1RA, and in the FLOW trial, only a small proportion were on SGLT2i at baseline [3, 4]. Multiple post hoc analyses suggest maintained efficacy of GLP‐1RAs in patients on SGLT2 inhibitors, but these data are limited by low power and selection bias [5, 6, 7, 8, 9, 10, 11, 12]. This gap is particularly relevant to adults with CKD and Type 2 diabetes (T2D), a population in whom GLP‐1RAs have shown kidney benefits earlier. Consequently, whether the addition of a GLP‐1RA confers complementary kidney benefits to patients already established on SGLT2i therapy remains unclear. To address this gap in the absence of completed head‐to‐head randomised trials, we leveraged a large, multi‐institutional electronic health record network. This study evaluates whether adding a GLP‐1RA to ongoing SGLT2i therapy reduces the progression of CKD and improves cardiovascular outcomes compared with continuing SGLT2i monotherapy in adults with CKD.

## 2 Methods

### 2.1 Data Source

This retrospective cohort study utilised data from the Global Collaborative Network within the TriNetX database, which compiled anonymized patient‐level information from electronic medical records provided by healthcare organizations (HCOs). An HCO typically includes academic medical centers, community hospitals, and outpatient clinics. The Global Collaborative Network comprised data from 152 HCOs, with the majority based in the USA.

The recorded data include patient demographics, encounters, diagnoses, procedures, medications, and laboratory measurements. The TriNetX platform employs standardised terminology mapping and preprocessing algorithms to ensure data quality and minimise missingness. The results were provided to investigators in aggregate, de‐identified form. The TriNetX platform and its data structure have been described in detail elsewhere [16].

This study was conducted in compliance with the Declaration of Helsinki and adhered to the Strengthening the Reporting of Observational Studies in Epidemiology (STROBE) guidelines [17]. Ethical review was not required, as all data were de‐identified and analysed in compliance with HIPAA regulations.

### 2.2 Study Population

The current study included adults aged ≥ 18 years diagnosed with CKD and treated with SGLT2i and GLP‐1RA or SGLT2i between 1 June 2020 and 31 December 2023. The CKD population was defined by the diagnosis code for CKD and at least one of the following criteria: eGFR < 60 mL/min/1.73 m^2^, a diagnosis of proteinuria, urine albumin/creatinine ratio (UACR) ≥ 30 mg/g or urine protein/creatinine ratio ≥ 150 mg/g (Figure 1). These clinical criteria were required to occur on or before the index date to ensure confirmation of CKD status prior to cohort entry.

**FIGURE 1:**
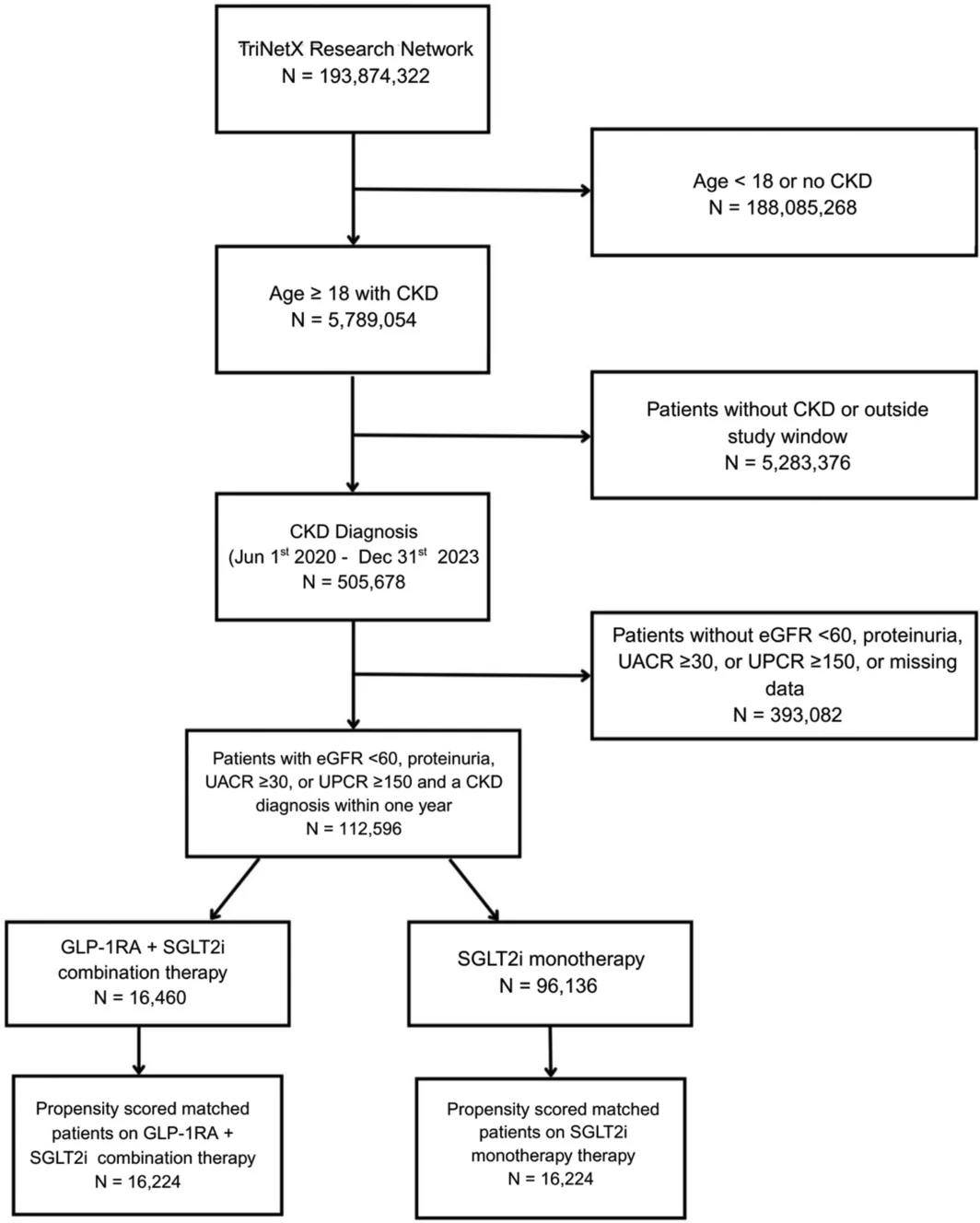
Consort diagram depicting the inclusion and exclusion criteria for patients on GLP‐1RA + SGLT2i combination therapy vs. SGLT2i monotherapy.

Patients were then categorised into two exposure groups: those receiving SGLT2i monotherapy (SGLT2i group) and those established on SGLT2i therapy who subsequently initiated a GLP‐1RA. The index date was defined as the date of the prescription of a GLP‐1RA. Patients were required to have active SGLT2i coverage (a prescription within the preceding 30 days) at the time of GLP‐1RA initiation. The combination cohort was defined as patients with active SGLT2i therapy who initiated a GLP‐1RA (Index Date). Active treatment was confirmed by the presence of an SGLT2i prescription within the 30 days preceding the GLP‐1RA initiation. The SGLT2i monotherapy cohort consisted of adults with CKD who received treatment with an SGLT2 inhibitor during the study period and had no previous recorded exposure to GLP‐1RA. The index date for this cohort was defined as the date of SGLT2 inhibitor initiation. This design ensures that both groups were at a comparable stage of established SGLT2i therapy at the time of cohort entry, aligning the ‘treatment decision point’ across both arms. Patients with existing CKD Stage 5, end‐stage renal disease (ESRD), or dialysis dependence on or before the index date were excluded to avoid misidentifying pre‐existing advanced kidney disease or kidney replacement therapy as incident kidney outcomes during follow‐up [18, 19]. Details of cohort definitions and coding algorithms are available in Tables S2 and S3.

To assess the consistency of treatment effects, we conducted subgroup analyses across clinically relevant categories. Subgroup analyses were stratified by T2D status (T2D vs. non‐T2D), obesity status (BMI ≥ 30 kg/m^2^ vs. BMI < 30 kg/m^2^), and heart failure (HF). Additional stratification was performed by CKD Stage 1–2 (ICD N18.1 or N18.2), Stage 3 (ICD N18.3), and Stage 4 (ICD N18.4), history of ischemic heart disease (IHD vs. no IHD), and age group (18–65 years vs. ≥ 65 years).

### 2.3 Covariates

We performed 1:1 propensity score matching (PSM) using a greedy nearest‐neighbour algorithm with a calliper width of 0.1. Propensity scores were estimated using logistic regression based on user‐defined baseline covariates assessed within 1 year prior to the index date, followed by matching patients from the smaller cohort with those from the larger cohort based on sufficiently similar propensity scores [20]. Demographic variables included age, sex, race, and ethnicity. Clinical characteristics incorporated into the model were Body Mass Index (BMI), systolic blood pressure, and key comorbidities including T2D, hypertension, HF, atrial fibrillation, atherosclerotic cardiovascular disease (ASCVD), and microvascular complications. Laboratory measures included the most recent values for HbA1c, eGFR, LDL‐cholesterol, and UACR. We also adjusted for concomitant medication use, including insulin, metformin, RAAS inhibitors, statins, diuretics, and other antihypertensives. Covariate balance was assessed using Standardised Mean Differences (SMD), with a threshold of < 0.1 indicating adequate balance. Detailed definitions and codes are provided in Table S4.

### 2.4 Prespecified Outcomes

The primary outcome was major adverse kidney events (MAKE), defined as a composite of all‐cause mortality, progression to end‐stage kidney disease (ESKD), dialysis, CKD Stage 5, or renal transplant. Secondary outcomes included major adverse cardiovascular events (MACE), defined as a composite of all‐cause mortality, acute myocardial infarction, stroke, and cardiac arrest. Safety endpoints included the incidence of gastrointestinal events, gallbladder and pancreatic disorders, severe hypoglycemia, genital mycotic infections, thyroid cancer, suicide attempts, palpitations, hypotension, dehydration, and progression to diabetic or hypertensive retinopathy. Safety endpoints were selected based on established and clinically relevant safety concerns previously evaluated for GLP‐1 receptor agonists and SGLT2 inhibitors [21, 22]. Outcome definitions were based on established clinical coding algorithms and definitions, incorporating relevant ICD‐10 and procedure codes, and were informed by previously published real‐world studies utilising the TriNetX platform, as detailed in Table S5 [23].

### 2.5 Statistical Analysis

Baseline characteristics were compared using SMDs post‐matching. We employed an intention‐to‐treat (ITT) approach for all analyses. Time‐to‐event outcomes were analyzed using the Kaplan–Meier method and Cox proportional hazards models to estimate hazard ratios (HR) and 95% confidence intervals (CI). The proportional hazards assumption was verified using Schoenfeld residuals. Patients were censored at the earliest of outcome occurrence, death, loss to follow‐up (defined as a gap in EMR activity > 1 year), or the study end date (December 31, 2023). We calculated relative risk reduction (RRR) based on 1 year survival probabilities [24]. *E*‐values were computed to quantify the potential impact of unmeasured confounding [25]. Pre‐specified subgroup analyses were performed, with heterogeneity assessed using Cochran's Q test (P‐interaction). All tests were two‐sided with a significance level of 0.05.

## 3 Results

### 3.1 Baseline Characteristics

The initial search identified 112 596 patients with CKD receiving SGLT2i therapy. Of these, 16 460 (14.6%) were incident users of a GLP‐1RA (combination group), while 96 136 (85.4%) remained on SGLT2i monotherapy. Before matching, patients in the combination group were younger, had a higher prevalence of obesity and dyslipidemia, and had greater utilisation of cardioprotective medications, including statins and RAAS inhibitors. Following 1:1 propensity score matching, the final analytic cohort comprised 32 448 patients (16 224 per group). The matching algorithm achieved excellent balance across all baseline covariates, including demographics, comorbidities, laboratory parameters, and medication use, with all SMD < 0.1 (Table S1).

### 3.2 Primary Outcome

Over the 12‐month follow‐up period, the addition of a GLP‐1RA to SGLT2i therapy was associated with a significant reduction in the risk of the primary composite outcome, MAKE (HR, 0.70 [95% CI, 0.65–0.74]; *p* < 0.001), compared with SGLT2i monotherapy. Analysis of individual components revealed that this benefit was driven by a reduction in progression to CKD Stage 5 (HR, 0.77 [95% CI, 0.66–0.89]) and incident dialysis/ESKD (HR, 0.83 [95% CI, 0.76–0.91]), and all‐cause mortality, which is reported separately below. No significant difference in renal transplantation incidence was observed (HR, 1.44 [95% CI, 0.51–4.04]) (Figure 2).

**FIGURE 2:**
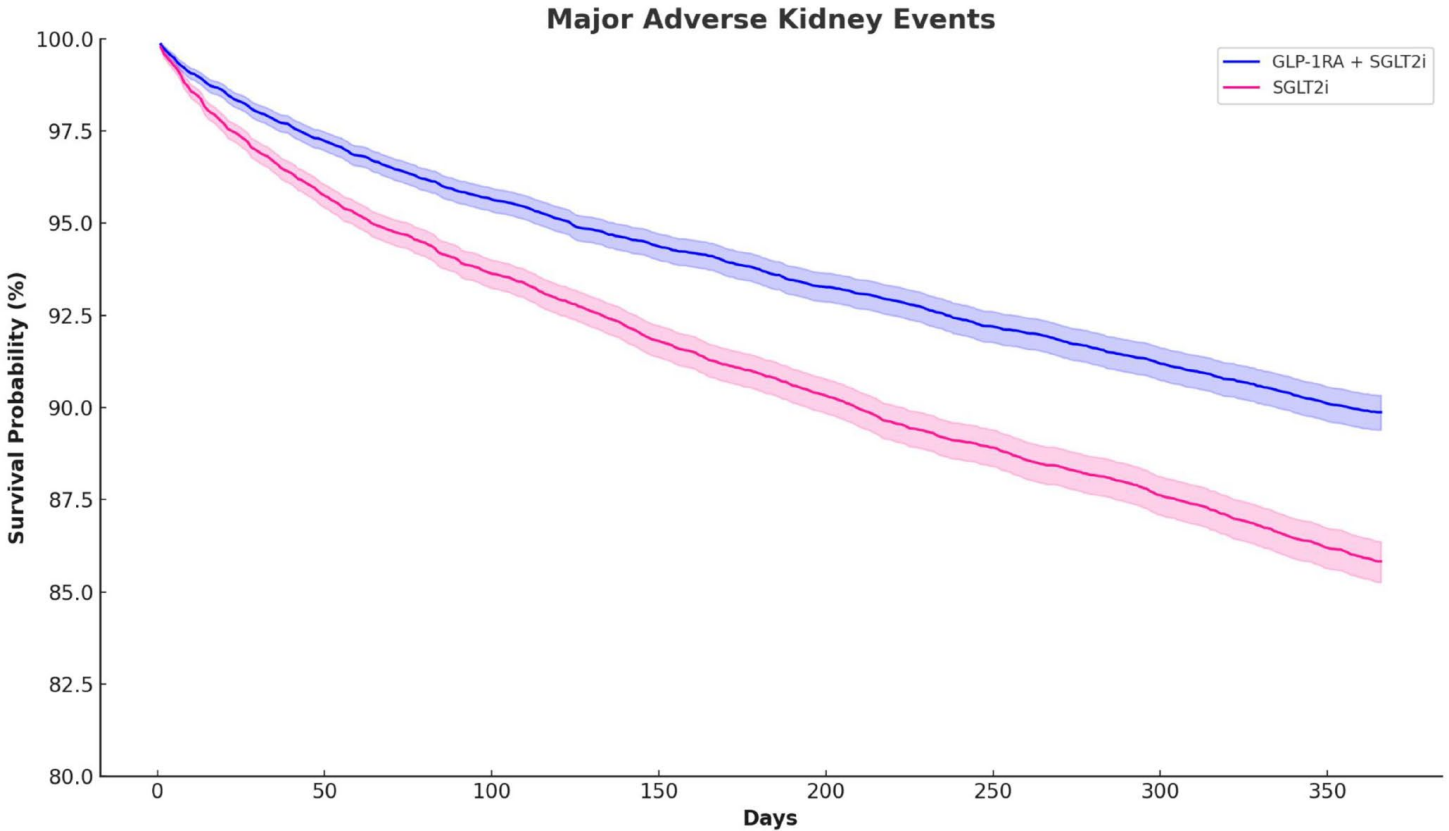
Kaplan–Meier curve for 12‐month incidence of MAKE in patients with CKD treated with combination therapy (GLP‐1RA and SGLT2i) versus monotherapy (SGLT2i alone).

Combination therapy was associated with a lower risk of all‐cause mortality (HR, 0.55 [95% CI, 0.50–0.61]; *p* < 0.001). The robustness of this association was supported by an *E*‐value of 2.38, suggesting that unmeasured confounding would need to be substantial to account for the observed effect size (Figure 3). Combination therapy was associated with a significant reduction for MACE (HR, 0.83 [95% CI, 0.78–0.87]; *p* < 0.001) compared to SGLT2i alone. This reduction was accompanied by lower all‐cause mortality, as described previously, and a reduced risk of cardiac arrest (HR, 0.58 [95% CI, 0.47–0.71]), while associations with acute myocardial infarction (HR, 0.95 [95% CI, 0.88–1.03]) and stroke (HR, 0.92 [95% CI, 0.85–1.01]) did not reach statistical significance (Figure 4). A summary of clinical outcomes comparing combination therapy with SGLT2i monotherapy is provided in Figure 5 and Table S6.

**FIGURE 3:**
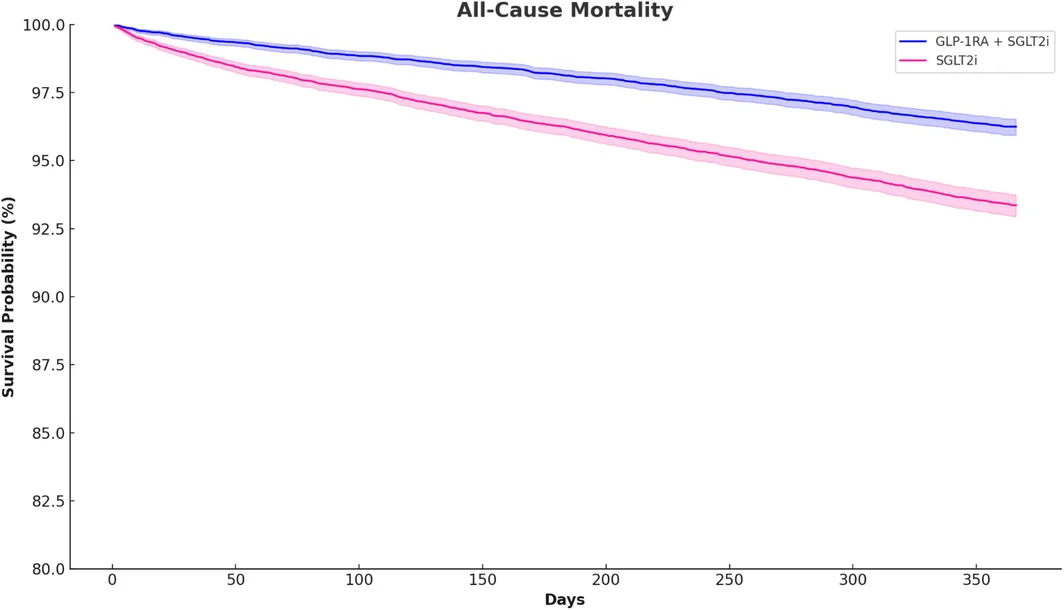
Kaplan–Meier curve for 12‐month incidence of all‐cause mortality in patients with CKD treated with combination therapy (GLP‐1RA and SGLT2i) monotherapy (SGLT2i alone).

**FIGURE 4:**
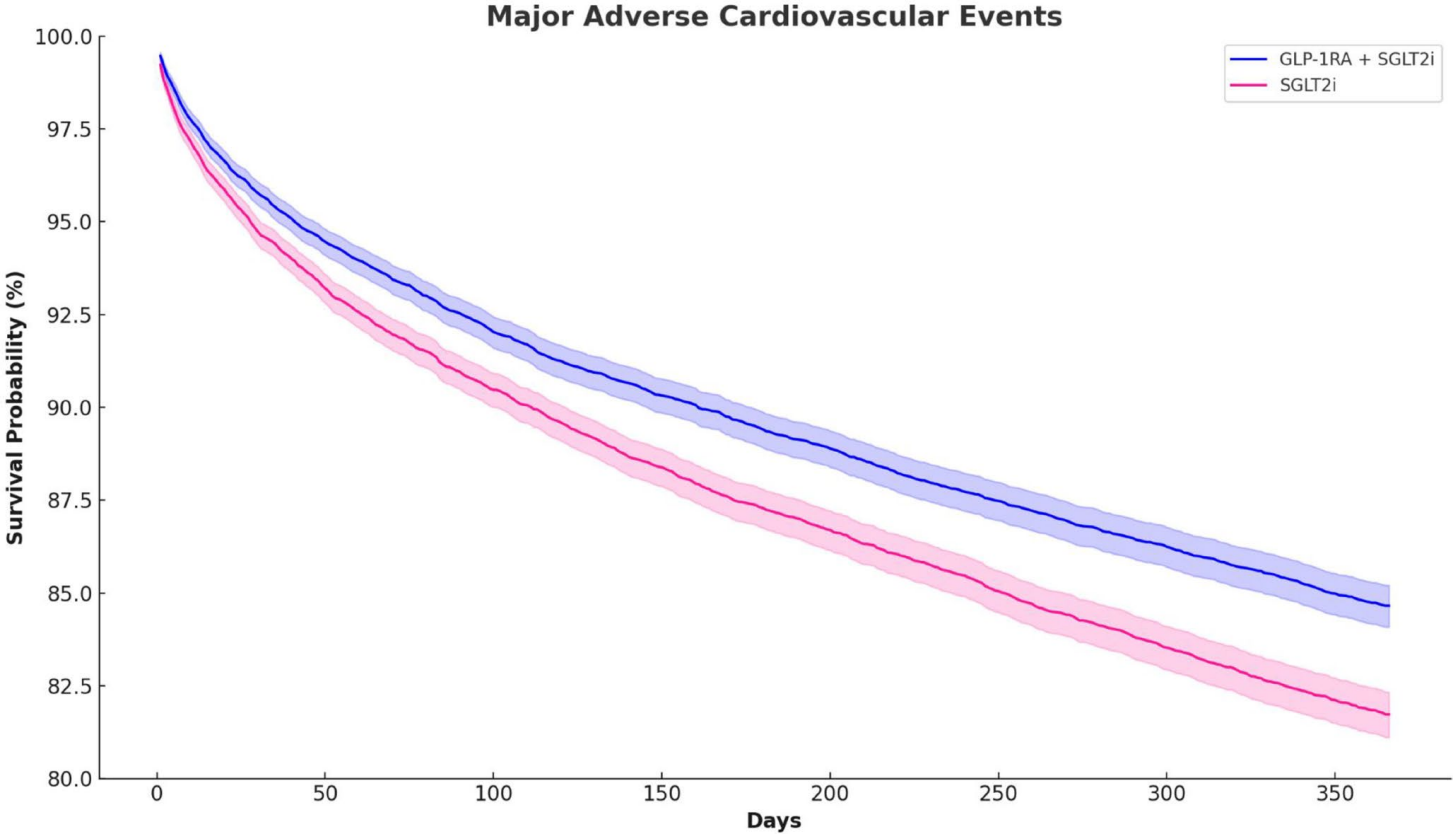
Kaplan–Meier curve for 12‐month incidence of MACE in patients with CKD treated with combination therapy (GLP‐1RA and SGLT2i) versus monotherapy (SGLT2i alone).

**FIGURE 5:**
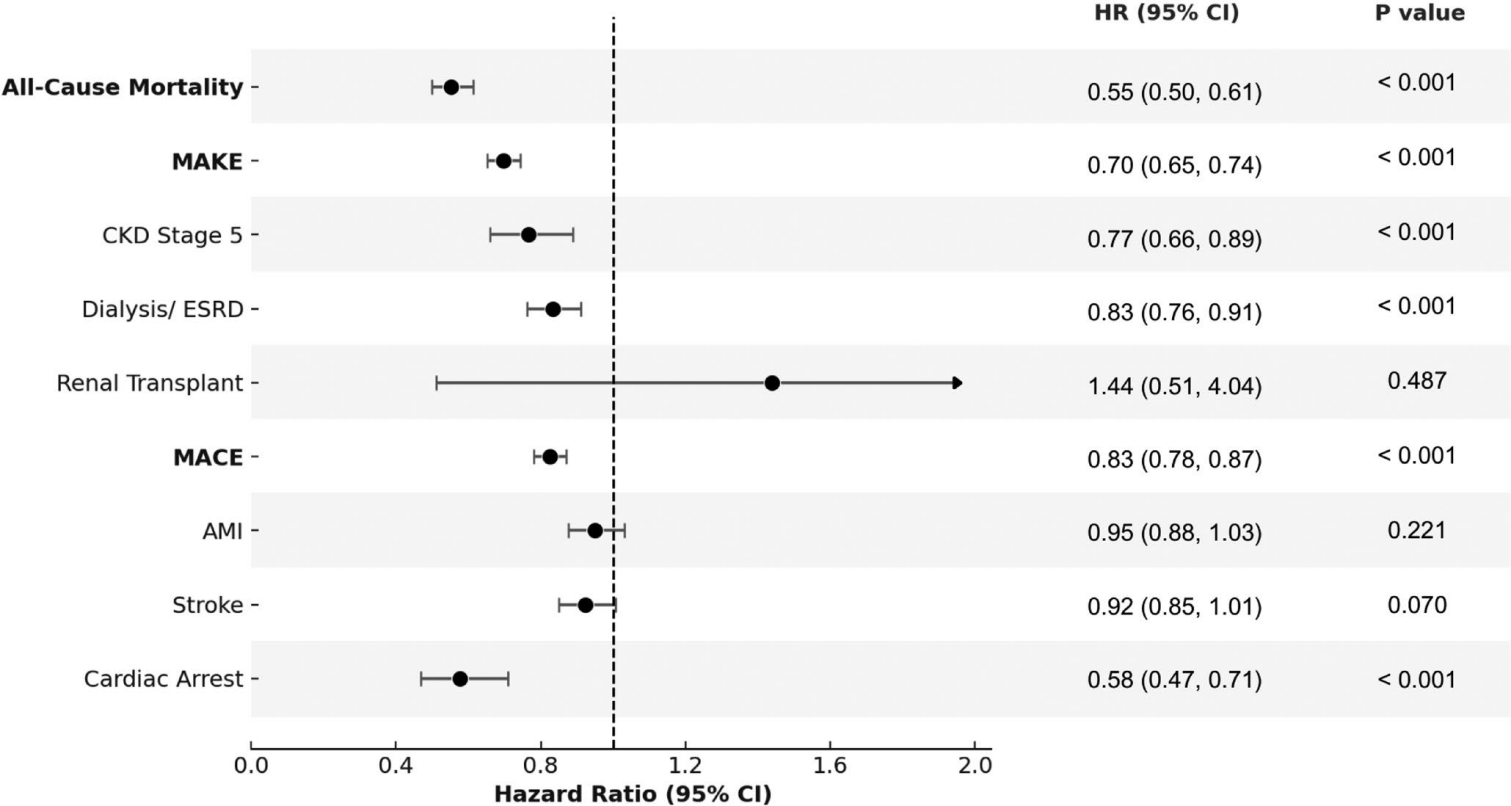
Clinical outcomes comparing patients on combination therapy (GLP‐1RA and SGLT2i) versus on monotherapy (SGLT2i).

### 3.3 Subgroup Analyses

The kidney and survival benefits of combination therapy were consistent across most clinically relevant subgroups, including age, diabetes status, and baseline kidney function. However, significant effect modification was observed by obesity status and HF history (*p*‐interaction < 0.05 for both). The reduction in all‐cause mortality was more pronounced in patients with obesity (HR, 0.49 vs. 0.63, *p*‐interaction = 0.021) than in those without obesity, and in patients with a history of HF (HR, 0.59 vs. HR, 0.79; *p*‐interaction = 0.026) than in those without. Similarly, the reduction for MAKE was significantly greater in the obesity subgroup (HR, 0.70 vs. 0.82; *p*‐interaction = 0.037) and among patients with macroalbuminuria (UACR ≥ 300 mg/g) (HR, 0.57 vs. 0.73 vs. 0.78; *p*‐interaction = 0.155) (Figures S1–S3).

### 3.4 Safety and Adverse Events

Safety analysis revealed a distinct risk profile for combination therapy. Consistent with the known adverse event profile of GLP‐1RAs, patients in the combination group had a higher risk of gastrointestinal symptoms (HR, 1.09 [95% CI, 1.04–1.13]) and progression of diabetic retinopathy (HR, 1.48 [95% CI, 1.40–1.57]). An increased risk of genital mycotic infections was also observed (HR, 1.29 [95% CI, 1.08–1.55]). Conversely, combination therapy was associated with significantly lower risks of several adverse events, including hypotension/syncope (HR, 0.92 [95% CI, 0.87–0.98]), dehydration (HR, 0.87 [95% CI, 0.79–0.95]), and palpitations (HR, 0.88 [95% CI, 0.83–0.93]). No significant difference in severe hypoglycemia was observed between groups (HR, 1.03 [95% CI, 0.90–1.18]; *p* = 0.63) (Table 1).

**TABLE 1:** Safety outcomes in patients on combination therapy (GLP‐1RA and SGLT2i) versus on monotherapy (SGLT2i).

	GLP‐1RA + SGLT2i (16 224)	SGLT2i (16 224)	Hazard ratio (95% CI)	*p*	*E*‐value (CI upper limit)
Gastrointestinal symptoms	4694 (28.94)	4192 (25.84)	1.09 (1.04, 1.13)	< 0.001	1.31 (1.40)
Urinary tract infection	1428 (8.80)	1538 (9.48)	0.89 (0.83, 0.95)	0.001	1.40 (1.22)
Hypoglycemia	436 (2.69)	403 (2.48)	1.03 (0.90, 1.18)	0.633	1.18 (1.50)
Suicide attempts	71 (0.44)	110 (0.68)	0.62 (0.46, 0.83)	0.001	2.15 (1.54)
Hypotension/Syncope	2014 (12.42)	2080 (12.82)	0.92 (0.87, 0.98)	0.010	1.31 (1.13)
Retinopathy progression	2958 (18.23)	1989 (12.26)	1.48 (1.40, 1.57)	< 0.001	1.95 (2.07)
Genital mycotic infection	285 (1.76)	211 (1.30)	1.29 (1.08, 1.55)	0.005	1.68 (2.04)
Dehydration/Volume depletion	877 (5.41)	963 (5.94)	0.87 (0.79, 0.95)	0.002	1.45 (1.23)
Palpitations	1957 (12.06)	2116 (13.04)	0.88 (0.83, 0.93)	< 0.001	1.42 (1.28)
Gallbladder and pancreatic disorders	1077 (6.64)	1234 (7.61)	0.83 (0.77, 0.90)	< 0.001	1.53 (1.36)
Thyroid cancer	74 (0.46)	73 (0.45)	0.98 (0.71, 1.35)	0.900	1.14 (1.77)

## 4 Discussion

In this large, multi‐institutional and multi‐national cohort study of adults with CKD, we found that the addition of a GLP‐1RA to ongoing SGLT2i therapy was associated with a significant reduction in the risk of major adverse kidney events, MACE, and all‐cause mortality compared with SGLT2i monotherapy alone. While the combination was broadly effective, we identified distinct high‐risk phenotypes that derived significantly greater benefit. Significant effect modification was observed based on cardiometabolic burden, with the greatest relative risk reduction occurring in patients with obesity, HF, and IHD. These findings suggest that dual incretin‐SGLT2 inhibition targets specific “residual risk” pathways, namely adiposity and established atherosclerosis that persist despite standard SGLT2i blockade.

While the FLOW trial recently established the renoprotective efficacy of semaglutide, and trials such as DAPA‐CKD and EMPA‐KIDNEY cemented SGLT2i as the standard of care, evidence regarding their combined effects has been limited to post hoc analyses and small physiologic studies [5, 6, 7, 26]. Our findings bridge this gap by demonstrating that these agents are likely synergistic and offer important renoprotective benefits. Mechanistically, SGLT2 inhibitors primarily reduce intraglomerular pressure via tubuloglomerular feedback (hemodynamic off‐loading) and induce metabolic flexibility through ketogenesis. In contrast, GLP‐1RAs exert direct anti‐inflammatory, antioxidant, and anti‐fibrotic effects by suppressing the NF‐κB pathway, while promoting additional natriuresis by inhibiting the proximal tubular NHE3, with commensurate reduction in visceral adiposity, a key driver of glomerular hyperfiltration and pro‐inflammatory cytokines [27]. By targeting these distinct non‐overlapping pathways (hemodynamic vs. inflammatory/metabolic), combination therapy addresses the “residual risk” that persists despite maximal SGLT2i blockade [28, 29].

A key novel insight from our study is the identification of clinical phenotypes that may warrant prioritised dual therapy. The amplified benefit in patients with BMI ≥ 30 kg/m^2^ aligns with the hypothesis that GLP‐1RAs effectively target “obesity‐related glomerulopathy” by reducing ectopic lipid accumulation and systemic inflammation. SGLT2 inhibitors primarily act through hemodynamic mechanisms that are less dependent on adiposity; the combination with a GLP‐1RA may provide complementary benefits for kidney and cardiovascular outcomes. This suggests that BMI could serve as a clinical biomarker for prioritising combination therapy [30, 31, 32].

The reduction in MACE, driven mainly by cardiac arrest and mortality, is consistent with the established anti‐atherosclerotic properties of GLP‐1RAs (LEADER, SUSTAIN‐6) and the volume‐control benefits of SGLT2 inhibitors [27, 33]. Interestingly, we observed pronounced benefits in patients with pre‐existing HF. While SGLT2 inhibitors are foundational for HF, recent evidence suggests GLP‐1RAs improve outcomes in HF with preserved ejection fraction (HFpEF) by reducing epicardial adipose tissue and left ventricular stiffness [34, 35]. Our data support the concept that dual therapy offers comprehensive cardioprotection: SGLT2i managing volume/hemodynamics and GLP‐1RA managing the inflammatory‐metabolic substrate of the myocardium [36, 37].

Our safety analysis provides a reassuring but cautionary profile. The increased risk of retinopathy progression (HR 1.48) replicates the signal seen in the SUSTAIN‐6 trial. This is believed to be a transient phenomenon driven by rapid glucose lowering rather than direct retinal toxicity. It highlights the critical need for ophthalmologic screening prior to initiating combination therapy, particularly in patients with high baseline HbA1c [38, 39]. Conversely, the reduction in hypotension and dehydration events with combination therapy is intriguing. This may reflect reduced need for loop diuretics due to improved glycemic and kidney outcomes, or differences in sodium handling between GLP‐1RA and SGLT2i, though this requires confirmation in dedicated physiologic studies [40].

These mechanistic pathways contextualise the directionally favourable but non‐significant trends seen in our T2D subgroup. Although our T2D subgroup did not reach statistical significance, large trials such as CREDENCE and DAPA‐CKD consistently show a 25%–40% reduction in kidney failure risk with SGLT2i alone [27]. The numerically lower risk estimates seen with combination therapy in our cohort, therefore, remain biologically plausible and may reflect limited power rather than a lack of effect.

This study has several limitations that warrant consideration. First, the exposure definitions used in this retrospective cohort design may introduce some potential for immortal‐time bias because the combination therapy cohort was defined by subsequent initiation of a GLP‐1RA among patients receiving SGLT2i therapy, whereas the monotherapy cohort was defined at SGLT2i initiation. To minimise the period between SGLT2i exposure and entry into the combination cohort, we required active SGLT2i use within 30 days before GLP‐1RA initiation; however, this design feature does not completely eliminate the possibility of non‐aligned time origins or residual immortal time. Since the retrospective observational design relies on electronic health records, there is also a risk of residual confounding, misclassification, and information bias. Second, exposure and outcome assessments may be impacted by uneven coding and incomplete documentation. However, our *E*‐value estimates and specificity analyses suggest that these biases are unlikely to fully explain the observed associations. Third, crucial treatment‐related variables, such as GLP‐1RA and SGLT2i dosage, titration, length of therapy, and medication adherence, were not adequately recorded and thus could not be assessed; these factors could affect safety and efficacy outcomes and warrant investigation in subsequent research. Fourth, although the overall direction of effects remained robust across sensitivity analyses, some confidence intervals were relatively wide, and follow‐up duration was short, limiting the accuracy of effect estimates and our ability to assess long‐term cardiovascular, renal, and safety outcomes. Fifth, even with strict matching and multiple bias‐mitigation techniques, causality cannot be determined from these data; large‐scale, prospective investigations are required to corroborate this. Sixth, outcome definitions were based on established clinical coding algorithms and previously published studies utilizing the TriNetX platform and were not independently validated in the present study. Study‐specific positive predictive values could not be determined because patient‐level chart review data are not available within the federated TriNetX database. Seventh, although baseline retinopathy and other microvascular complications were included as covariates in the propensity score model, the TriNetX database does not provide sufficient granularity to reliably distinguish retinopathy severity, including proliferative versus non‐proliferative disease. Therefore, we could not adequately assess the safety of dual GLP‐1RA and SGLT2i therapy specifically among patients with pre‐existing proliferative diabetic retinopathy. Lastly, the TriNetX database does not provide information regarding the specific clinical indication or rationale for prescribing SGLT‐2 inhibitors or GLP‐1 receptor agonists, particularly among patients with advanced CKD.

## 5 Conclusions

The addition of a GLP‐1RA to ongoing SGLT2i therapy is associated with a substantial reduction in the risk of kidney disease progression and cardiovascular events in a real‐world setting. These benefits appear most pronounced in patients with obesity and HF, suggesting distinct phenotypes that may benefit most from dual therapy. While confirmatory randomised trials are awaited, these data support the early adoption of combination therapy to address the high residual risk in patients with CKD.

## Funding

This research is funded by the National Institute for Health and Care Research (NIHR) Leicester Biomedical Research Centre (BRC). The views expressed are those of the authors and not necessarily those of the NIHR or the Department of Health and Social Care. Muhammad Rashid is funded by an NIHR DSE award (NIHR304156) and Academy of Medical Sciences grant (SGL025/1064). Gerry P. McCann receives support from the NIHR (Senior Investigator award) and the BHF Leicester Centre for Research Excellence.

## Disclosure

The authors have nothing to report.

## Ethics Statement

The authors have nothing to report.

## Conflicts of Interest

Kamlesh Khunti has acted as a consultant, speaker or received grants for investigator‐initiated studies for Abbott, Astra Zeneca, Bayer, Hikma, Novo Nordisk, Sanofi‐Aventis, Servier, Lilly, and Merck Sharp & Dohme, Boehringer Ingelheim, Oramed Pharmaceuticals, Pfizer, Roche, Daiichi‐Sankyo, Applied Therapeutics, Embecta, Nestle Health Science, and Adelphi. The other authors declare no conflicts of interest.

## Supporting information


**Table S1:** Baseline characteristics before and after propensity score matching.
**Table S2:** Eligibility criteria for patients on combination therapy (GLP‐1RA and SGLT2i). This query was run on the network Global Collaborative Network with 152 HCO(s) queried and 151 HCO(s) responded. A total of 83 provider(s) responded with patients. The final cohort included 16 460 patients who matched the query criteria listed in the table above.
**Table S3:** Eligibility criteria for patients in the monotherapy (SGLT2i alone). This query was run on the network Global Collaborative Network with 152 HCO(s) queried and 150 HCO(s) responded. A total of 110 provider(s) responded with patients. The final cohort included 96 136 patients who matched the query criteria listed in the table above.
**Table S4:** Demographics, diagnostic, laboratory, and medication codes used in definition of covariates.
**Table S5:** Outcome Definitions and Analysis Specifications. This table outlines the operational definitions for each outcome and the corresponding analysis parameters. For outcomes defined using multiple terms, the presence of at least one term is sufficient to meet the definition criteria.
**Table S6:** Clinical outcomes comparing patients on combination therapy (GLP‐1RA and SGLT2i) versus on monotherapy (SGLT2i).
**Figure S1:** Subgroup analysis of hazard ratios for MAKE.
**Figure S2:** Subgroup analysis of hazard ratios for all‐cause mortality.
**Figure S3:** Subgroup analysis of hazard ratios for MACE.

## Data Availability

The data analysed in this study were obtained from the TriNetX Global Collaborative Network (https://trinetx.com/), a federated research network comprising electronic health record data from participating healthcare organizations. The underlying patient‐level data are not publicly available due to data privacy, security, and governance restrictions. Researchers may access the TriNetX platform and conduct analyses using available datasets subject to TriNetX data‐use policies, participating healthcare organization requirements, and applicable governance procedures.
