# Supplementary material for: Effectiveness of Adding GLP‐1 Receptor Agonists to SGLT2 Inhibitor Therapy in Chronic Kidney Disease: A Population Based Study
Source: Diabetes Obes Metab. 2026 Sep 27;28(12):11755–64. doi: 10.1111/dom.71375 (PMC13730978; doi:10.1111/dom.71375)
Supplement: Supplementary file 1 [file DOM-28-11755-s001.docx]

**Supplementary Material**

**Supplementary Table 1.** Baseline Characteristics Before and After Propensity Score Matching.

|  | **Data prior to matching** | | | **Propensity Matched Cohorts** | | |
| --- | --- | --- | --- | --- | --- | --- |
|  | **GLP-1RA +** **SGLT2i (16,460)** | **SGLT2i**  **(96,136)** | **Std Diff.** | **GLP-1RA + SGLT2i**  **(16,224)** | **SGLT2i alone (16,224)** | **Std Diff** |
| **Demographics** |  |  |  |  |  |  |
| Age (mean ± SD) | 68.5 ± 10.7 | 72.7 ± 12.1 | 0.362 | 68.7 ± 10.6 | 68.6 ± 12.2 | 0.011 |
| White | 9,400 (57.1) | 51,293 (53.4) | 0.072 | 9,237 (56.9) | 9,323 (57.5) | 0.011 |
| Female | 6,782 (41.2) | 34,974 (36.4) | 0.093 | 6,648 (41.0) | 6,676 (41.1) | 0.004 |
| Black or African American | 3,686 (22.4) | 19,781 (20.6) | 0.062 | 3,622 (22.3) | 3,544 (21.8) | 0.012 |
| Asian | 1,150 (7.0) | 8,084 (8.4) | 0.057 | 1,147 (7.1) | 1,151 (7.1) | 0.001 |
| **Comorbidities** |  |  |  |  |  |  |
| Diabetes Mellitus | 15,468 (94.0) | 63,865 (66.4) | 0.737 | 15,232 (93.9) | 15,134 (93.3) | 0.025 |
| Dyslipidemia | 13,375 (81.3) | 66,910 (69.6) | 0.273 | 13,152 (81.1) | 12,853 (79.2) | 0.046 |
| Hypertension | 14,947 (90.8) | 82,750 (86.1) | 0.148 | 14,728 (90.8) | 14,410 (88.8) | 0.065 |
| Heart Failure | 5,387 (32.7) | 48,373 (50.3) | 0.363 | 5,378 (33.1) | 5,253 (32.4) | 0.016 |
| Acute Myocardial Infarction | 1,265 (7.7) | 12,677 (13.2) | 0.181 | 1,263 (7.8) | 1,187 (7.3) | 0.018 |
| Atrial Fibrillation and Flutter | 2,974 (18.1) | 29,287 (30.5) | 0.292 | 2,969 (18.3) | 2,836 (17.5) | 0.021 |
| Cerebrovascular Diseases | 2,116 (12.9) | 14,618 (15.2) | 0.068 | 2,107 (13.0) | 2,048 (12.6) | 0.011 |
| Peripheral Vascular Disease | 1,632 (9.9) | 9,538 (9.9) | <0.001 | 1,619 (10.0) | 1,621 (10.0) | <0.001 |
| Anemia | 4,510 (27.4) | 31,643 (32.9) | 0.120 | 4,481 (27.6) | 4,394 (27.1) | 0.012 |
| Chronic Lower Respiratory Diseases | 3,623 (22.0) | 23,433 (24.4) | 0.056 | 3,580 (22.1) | 3,558 (21.9) | 0.003 |
| Inflammatory Liver Disease | 360 (2.2) | 1,407 (1.5) | 0.054 | 351 (2.2) | 329 (2.0) | 0.009 |
| Liver Cirrhosis | 504 (3.1) | 3,130 (3.3) | 0.011 | 499 (3.1) | 490 (3.0) | 0.003 |
| All Neoplasms | 3,861 (23.5) | 22,181 (23.1) | 0.009 | 3,796 (23.4) | 3,678 (22.7) | 0.017 |
| Neoplasms |  |  |  |  |  |  |
| Respiratory Organs | 109 (0.7) | 1,200 (1.2) | 0.060 | 109 (0.7) | 119 (0.7) | 0.007 |
| Urinary Tract | 304 (1.8) | 2,015 (2.1) | 0.018 | 301 (1.9) | 293 (1.8) | 0.004 |
| Digestive Organs | 229 (1.4) | 1,814 (1.9) | 0.039 | 228 (1.4) | 207 (1.3) | 0.011 |
| Hematologic | 271 (1.6) | 2,446 (2.5) | 0.063 | 270 (1.7) | 258 (1.6) | 0.006 |
| Breast | 269 (1.6) | 1,485 (1.5) | 0.007 | 265 (1.6) | 265 (1.6) | <0.001 |
| Male Genital Organs | 377 (2.3) | 2,918 (3.0) | 0.046 | 375 (2.3) | 385 (2.4) | 0.004 |
| Ill-defined or Secondary | 182 (1.1) | 1,941 (2.0) | 0.074 | 181 (1.1) | 184 (1.1) | 0.002 |
| Dementia | 224 (1.4) | 2,834 (2.9) | 0.109 | 223 (1.4) | 225 (1.4) | 0.001 |
| Systemic Connective Tissue Disease | 354 (2.2) | 2,718 (2.8) | 0.043 | 351 (2.2) | 332 (2.0) | 0.008 |
| Rheumatoid Arthritis | 367 (2.2) | 2,144 (2.2) | <0.001 | 365 (2.2) | 346 (2.1) | 0.008 |
| **Medications** |  |  |  |  |  |  |
| HMG-CoA Reductase Inhibitors | 11,463 (69.6) | 57,526 (59.8) | 0.206 | 11,261 (69.4) | 10,958 (67.5) | 0.04 |
| Fibrates | 1,103 (6.7) | 3,253 (3.4) | 0.152 | 1,062 (6.5) | 999 (6.2) | 0.016 |
| Ezetimibe | 1,074 (6.5) | 4,639 (4.8) | 0.074 | 1,052 (6.5) | 999 (6.2) | 0.013 |
| Diuretics | 8,971 (54.5) | 57,583 (59.9) | 0.109 | 8,874 (54.7) | 8,695 (53.6) | 0.022 |
| Beta-Blockers | 9,000 (54.7) | 56,590 (58.9) | 0.085 | 8,912 (54.9) | 8,695 (53.6) | 0.027 |
| Calcium Channel Blockers | 6,357 (38.6) | 37,698 (39.2) | 0.012 | 6,306 (38.9) | 6,166 (38.0) | 0.018 |
| ACE Inhibitors | 4,658 (28.3) | 21,896 (22.8) | 0.127 | 4,567 (28.1) | 4,430 (27.3) | 0.019 |
| Angiotensin II Inhibitor | 6,286 (38.2) | 37,837 (39.4) | 0.024 | 6,208 (38.3) | 6,018 (37.1) | 0.024 |
| Insulin | 10,059 (61.1) | 35,461 (36.9) | 0.499 | 9,827 (60.6) | 9,695 (59.8) | 0.017 |
| **Laboratory Tests or Examinations** |  |  |  |  |  |  |
| eGFR ≥45 mL/min/1.73 m^2^ | 14,217 (86.4) | 85,674 (89.1) | 0.084 | 14,095 (86.9) | 13,785 (85.0) | 0.055 |
| Hb ≥12 g/dL | 12,399 (75.3) | 78,162 (81.3) | 0.145 | 12,305 (75.8) | 12,081 (74.5) | 0.032 |
| HbA1c ≥7% | 12,967 (78.8) | 61,659 (64.1) | 0.329 | 12,794 (78.9) | 12,653 (78.0) | 0.021 |
| BMI ≥30 kg/m^2^ | 12,210 (74.2) | 66,336 (69.0) | 0.115 | 12,004 (74.0) | 11,828 (72.9) | 0.025 |
| LDL ≥190 mg/dL | 10,709 (65.1) | 55,868 (58.1) | 0.143 | 10,569 (65.1) | 10,371 (63.9) | 0.026 |

| Supplementary Table 2. Eligibility criteria for patients on combination therapy (GLP-1RA and SGLT2i). This query was run on the network Global Collaborative Network with 152 HCO(s) queried and 151 HCO(s) responded. A total of 83 provider(s) responded with patients. The final cohort included 16,460 patients who matched the query criteria listed in the table above. | | | | | |
| --- | --- | --- | --- | --- | --- |
| Ungrouped terms | | | | | |
|  | must have |  | demographics | Age | Age (at least 18 years (most recent occurrence)) |
|  |  | and | medication | NLM:ATC:A10BJ | Glucagon-like peptide-1 (GLP-1) analogues |
|  |  | and | medication | NLM:ATC:A10BK | Sodium-glucose co-transporter 2 (SGLT2) inhibitors |
|  |  | and | diagnosis | UMLS:ICD10CM:N18 | Chronic kidney disease (CKD) |
| Group 1 | | | | | |
|  | **Group 1A** | | | | |
|  | must have |  | medication | NLM:ATC:A10BK | Sodium-glucose co-transporter 2 (SGLT2) inhibitors |
|  | date constraint | | The terms in this group occurred between Jun 1, 2020 and Dec 31, 2023 | | |
|  | event relationship | | Any instance of Group 1B occurred within 1 month on or after any instance of Group 1A | | |
|  | **Group 1B** | | | | |
|  | must have |  | medication | NLM:ATC:A10BJ | Glucagon-like peptide-1 (GLP-1) analogues |
| Group 2 | | | | | |
|  | **Group 2A** | | | | |
|  | must have |  | medication | NLM:ATC:A10BJ | Glucagon-like peptide-1 (GLP-1) analogues |
|  |  | and | medication | NLM:ATC:A10BK | Sodium-glucose co-transporter 2 (SGLT2) inhibitors |
|  | date constraint | | The terms in this group occurred between Jun 1, 2020 and Dec 31, 2023 | | |
|  | event relationship | | Any instance of Group 2B occurred within 1 year on or before the first instance of Group 2A | | |
|  | **Group 2B** | | | | |
|  | must have |  | diagnosis | UMLS:ICD10CM:N18 | Chronic kidney disease (CKD) |
| Group 3 | | | | | |
|  | **Group 3A** | | | | |
|  | must have |  | medication | NLM:ATC:A10BJ | Glucagon-like peptide-1 (GLP-1) analogues |
|  |  | and | medication | NLM:ATC:A10BK | Sodium-glucose co-transporter 2 (SGLT2) inhibitors |
|  | date constraint | | The terms in this group occurred between Jun 1, 2020 and Dec 31, 2023 | | |
|  | event relationship | | Any instance of Group 3B occurred on or before any instance of Group 3A | | |
|  | **Group 3B** | | | | |
|  | must have | any of | laboratory | TNX:8001 | Glomerular filtration rate/1.73 sq M.predicted [Volume Rate/Area] in Serum, Plasma or Blood by Creatinine-based formula (MDRD) (at most 60.00 mL/min/{1.73_m2}) |
|  |  |  | laboratory | UMLS:LNC:62238-1 | Glomerular filtration rate/1.73 sq M.predicted [Volume Rate/Area] in Serum, Plasma or Blood by Creatinine-based formula (CKD-EPI) (at most 60.00 mL/min/{1.73_m2}) |
|  |  |  | laboratory | UMLS:LNC:35591-7 | Creatinine renal clearance predicted by Cockcroft-Gault formula (at most 60.00 mL/min) |
|  |  |  | diagnosis | UMLS:ICD10CM:R80 | Proteinuria |
|  |  |  | laboratory | TNX:LG34557-5 | Albumin/Creatinine [Mass ratio] in Urine (at least 30.00 mg/g{creat}) |
|  |  |  | laboratory | TNX:LG34791-0 | Protein/Creatinine [Mass ratio] in Urine (at least 150.00 mg/g{creat}) |

**Supplementary Table 3**. Eligibility criteria for patients in the monotherapy (SGLT2i alone). This query was run on the network Global Collaborative Network with 152 HCO(s) queried and 150 HCO(s) responded. A total of 110 provider(s) responded with patients. The final cohort included 96,136 patients who matched the query criteria listed in the table above.

| Ungrouped terms | | | | | |
| --- | --- | --- | --- | --- | --- |
|  | must have |  | demographics | Age | Age (at least 18 years (most recent occurrence)) |
|  |  | and | diagnosis | UMLS:ICD10CM:N18 | Chronic kidney disease (CKD) |
|  |  | and | medication | NLM:ATC:A10BK | Sodium-glucose co-transporter 2 (SGLT2) inhibitors |
|  | cannot have |  | medication | NLM:ATC:A10BJ | Glucagon-like peptide-1 (GLP-1) analogues |
| Group 1 | | | | | |
|  | **Group 1A** | | | | |
|  | must have |  | medication | NLM:ATC:A10BK | Sodium-glucose co-transporter 2 (SGLT2) inhibitors |
|  | date constraint | | The terms in this group occurred between Jun 1, 2020 and Dec 31, 2023 | | |
|  | event relationship | | Any instance of Group 1B occurred within 1 year on or before the first instance of Group 1A | | |
|  | **Group 1B** | | | | |
|  | must have |  | diagnosis | UMLS:ICD10CM:N18 | Chronic kidney disease (CKD) |
| Group 2 | | | | | |
|  | **Group 2A** | | | | |
|  | must have |  | medication | NLM:ATC:A10BK | Sodium-glucose co-transporter 2 (SGLT2) inhibitors |
|  | date constraint | | The terms in this group occurred between Jun 1, 2020 and Dec 31, 2023 | | |
|  | event relationship | | Any instance of Group 2B occurred on or before any instance of Group 2A | | |
|  | **Group 2B** | | | | |
|  | must have | any of | laboratory | TNX:8001 | Glomerular filtration rate/1.73 sq M.predicted [Volume Rate/Area] in Serum, Plasma or Blood by Creatinine-based formula (MDRD) (at most 60.00 mL/min/{1.73_m2}) |
|  |  |  | laboratory | UMLS:LNC:62238-1 | Glomerular filtration rate/1.73 sq M.predicted [Volume Rate/Area] in Serum, Plasma or Blood by Creatinine-based formula (CKD-EPI) (at most 60.00 mL/min/{1.73_m2}) |
|  |  |  | laboratory | UMLS:LNC:35591-7 | Creatinine renal clearance predicted by Cockcroft-Gault formula (at most 60.00 mL/min) |
|  |  |  | diagnosis | UMLS:ICD10CM:R80 | Proteinuria |
|  |  |  | laboratory | TNX:LG34557-5 | Albumin/Creatinine [Mass ratio] in Urine (at least 30.00 mg/g{creat}) |
|  |  |  | laboratory | TNX:LG34791-0 | Protein/Creatinine [Mass ratio] in Urine (at least 150.00 mg/g{creat}) |

**Supplementary Table 4.** Demographics, diagnostic, laboratory, and medication codes used in definition of covariates.

| Category | Code | Description |  |
| --- | --- | --- | --- |
| Demographics | Age | Current Age | |
| Demographics | 2106-3 | White | |
| Demographics | F | Female | |
| Demographics | 2054-5 | Black or African American | |
| Demographics | 2028-9 | Asian | |
| Diagnosis | E08-E13 | Diabetes mellitus | |
| Diagnosis | E78 | Disorders of lipoprotein metabolism and other lipidemias | |
| Diagnosis | I10-I1A | Hypertensive diseases | |
| Diagnosis | I50 | Heart failure | |
| Diagnosis | I21 | Acute myocardial infarction | |
| Diagnosis | I48 | Atrial fibrillation and flutter | |
| Diagnosis | I60-I69 | Cerebrovascular diseases | |
| Diagnosis | I73.9 | Peripheral vascular disease, unspecified | |
| Diagnosis | D60-D64 | Aplastic and other anemias and other bone marrow failure syndromes | |
| Diagnosis | J40-J4A | Chronic lower respiratory diseases | |
| Diagnosis | K75 | Other inflammatory liver diseases | |
| Diagnosis | K74 | Fibrosis and cirrhosis of liver | |
| Diagnosis | C30-C39 | Malignant neoplasms of respiratory and intrathoracic organs | |
| Diagnosis | C64-C68 | Malignant neoplasms of urinary tract | |
| Diagnosis | C15-C26 | Malignant neoplasms of digestive organs | |
| Diagnosis | C81-C96 | Malignant neoplasms of lymphoid, hematopoietic and related tissue | |
| Diagnosis | C50-C50 | Malignant neoplasms of breast (C50) | |
| Diagnosis | C60-C63 | Malignant neoplasms of male genital organs | |
| Diagnosis | C76-C80 | Malignant neoplasms of ill-defined, other secondary and unspecified sites | |
| Diagnosis | F03 | Unspecified dementia | |
| Diagnosis | M30-M36 | Systemic connective tissue disorders | |
| Diagnosis | M06 | Other rheumatoid arthritis | |
| Diagnosis | C00-D49 | Neoplasms | |
| Medication | C10AA | HMG CoA reductase inhibitors | |
| Medication | C10AB | Fibrates | |
| Medication | 341248 | ezetimibe | |
| Medication | C03 | DIURETICS | |
| Medication | C07 | BETA BLOCKING AGENTS | |
| Medication | C08 | CALCIUM CHANNEL BLOCKERS | |
| Medication | CV800 | ACE INHIBITORS | |
| Medication | CV805 | ANGIOTENSIN II INHIBITOR | |
| Medication | HS501 | INSULIN | |
| Laboratory | 8001 | Glomerular filtration rate/1.73 sq M.predicted [Volume Rate/Area] in Serum, Plasma or Blood by Creatinine-based formula (MDRD) | |
| Laboratory | 9014 | Hemoglobin [Mass/volume] in Blood | |
| Laboratory | 9037 | Hemoglobin A1c/Hemoglobin.total in Blood | |
| Laboratory | 9083 | BMI | |
| Laboratory | 9002 | Cholesterol in LDL [Mass/volume] in Serum or Plasma | |
| Laboratory | LG307-5 | Albumin [Mass/volume] in Urine | |

**Supplementary Table 5.** Outcome Definitions and Analysis Specifications. This table outlines the operational definitions for each outcome and the corresponding analysis parameters. For outcomes defined using multiple terms, the presence of at least one term is sufficient to meet the definition criteria.

| MAKE | | | | |
| --- | --- | --- | --- | --- |
|  | **Outcome definition** | | | |
|  | | Diagnosis | UMLS:ICD10CM:N18.5 | Chronic kidney disease, stage 5 |
|  | | Diagnosis | UMLS:ICD10CM:N18.6 | End stage renal disease |
|  | | Diagnosis | UMLS:ICD10CM:N19 | Unspecified kidney failure |
|  | | Diagnosis | UMLS:ICD10CM:Z99.2 | Dependence on renal dialysis |
|  | | Procedure | UMLS:CPT:1012740 | Dialysis Services and Procedures |
|  | | Procedure | UMLS:CPT:1029674 | Dialysis Circuit Procedures |
|  | | Procedure | UMLS:CPT:1008098 | Renal Transplantation Procedures |
|  | | Demographics | Deceased | Deceased |
|  | **Settings for the performed analyses** | | | |
|  | | Risk analysis | | including patients with outcome prior to the time window |
|  | | Kaplan - Meier survival analysis | | including patients with outcome prior to the time window |
| All-cause death | | | | |
|  | **Outcome definition** | | | |
|  | | Diagnosis | UMLS:ICD10CM:R99-R99 | Ill-defined and unknown cause of mortality (R99) |
|  | | Demographics | Deceased | Deceased |
|  | **Settings for the performed analyses** | | | |
|  | | Kaplan - Meier survival analysis | | including patients with outcome prior to the time window |
|  | | Risk analysis | | including patients with outcome prior to the time window |
| Major adverse cardiac events | | | | |
|  | **Outcome definition** | | | |
|  | | Diagnosis | UMLS:ICD10CM:I21 | Acute myocardial infarction |
|  | | Diagnosis | UMLS:ICD10CM:I61 | Nontraumatic intracerebral hemorrhage |
|  | | Diagnosis | UMLS:ICD10CM:I62 | Other and unspecified nontraumatic intracranial hemorrhage |
|  | | Diagnosis | UMLS:ICD10CM:I63 | Cerebral infarction |
|  | | Diagnosis | UMLS:ICD10CM:I46.2 | Cardiac arrest due to underlying cardiac condition |
|  | | Diagnosis | UMLS:ICD10CM:I46.9 | Cardiac arrest, cause unspecified |
|  | | Demographics | Deceased | Deceased |
|  | **Settings for the performed analyses** | | | |
|  | | Risk analysis | | including patients with outcome prior to the time window |
|  | | Kaplan - Meier survival analysis | | including patients with outcome prior to the time window |
| GI symptoms | | | | |
|  | **Outcome definition** | | | |
|  | | Diagnosis | UMLS:ICD10CM:R10-R19 | Symptoms and signs involving the digestive system and abdomen |
|  | **Settings for the performed analyses** | | | |
|  | | Risk analysis | | including patients with outcome prior to the time window |
|  | | Kaplan - Meier survival analysis | | including patients with outcome prior to the time window |
| UTI | | | | |
|  | **Outcome definition** | | | |
|  | | Diagnosis | UMLS:ICD10CM:N39.0 | Urinary tract infection, site not specified |
|  | **Settings for the performed analyses** | | | |
|  | | Risk analysis | | including patients with outcome prior to the time window |
|  | | Kaplan - Meier survival analysis | | including patients with outcome prior to the time window |
| hypoglycemia | | | | |
|  | **Outcome definition** | | | |
|  | | Diagnosis | UMLS:ICD10CM:E16.0 | Drug-induced hypoglycemia without coma |
|  | | Diagnosis | UMLS:ICD10CM:E16.1 | Other hypoglycemia |
|  | | Diagnosis | UMLS:ICD10CM:E16.2 | Hypoglycemia, unspecified |
|  | **Settings for the performed analyses** | | | |
|  | | Risk analysis | | including patients with outcome prior to the time window |
|  | | Kaplan - Meier survival analysis | | including patients with outcome prior to the time window |
| suicidal attempts | | | | |
|  | **Outcome definition** | | | |
|  | | Diagnosis | UMLS:ICD10CM:R45.851 | Suicidal ideations |
|  | | Diagnosis | UMLS:ICD10CM:T14.91 | Suicide attempt |
|  | **Settings for the performed analyses** | | | |
|  | | Risk analysis | | including patients with outcome prior to the time window |
|  | | Kaplan - Meier survival analysis | | including patients with outcome prior to the time window |
| hypotension/syncope | | | | |
|  | **Outcome definition** | | | |
|  | | Diagnosis | UMLS:ICD10CM:I95 | Hypotension |
|  | | Diagnosis | UMLS:ICD10CM:R55 | Syncope and collapse |
|  | **Settings for the performed analyses** | | | |
|  | | Risk analysis | | including patients with outcome prior to the time window |
|  | | Kaplan - Meier survival analysis | | including patients with outcome prior to the time window |
| Retinopathy progression | | | | |
|  | **Outcome definition** | | | |
|  | | Diagnosis | UMLS:ICD10CM:E10.3 | Type 1 diabetes mellitus with ophthalmic complications |
|  | | Diagnosis | UMLS:ICD10CM:E11.3 | Type 2 diabetes mellitus with ophthalmic complications |
|  | | Diagnosis | UMLS:ICD10CM:E13.3 | Other specified diabetes mellitus with ophthalmic complications |
|  | | Diagnosis | UMLS:ICD10CM:H35.039 | Hypertensive retinopathy, unspecified eye |
|  | | Diagnosis | UMLS:ICD10CM:H34.81 | Central retinal vein occlusion |
|  | | Diagnosis | UMLS:ICD10CM:H34.83 | Tributary (branch) retinal vein occlusion |
|  | | Diagnosis | UMLS:ICD10CM:H35.6 | Retinal hemorrhage |
|  | | Diagnosis | UMLS:ICD10CM:H35.8 | Other specified retinal disorders |
|  | | Diagnosis | UMLS:ICD10CM:H35.9 | Unspecified retinal disorder |
|  | **Settings for the performed analyses** | | | |
|  | | Risk analysis | | including patients with outcome prior to the time window |
|  | | Kaplan - Meier survival analysis | | including patients with outcome prior to the time window |
| Genital mycotic infections | | | | |
|  | **Outcome definition** | | | |
|  | | Diagnosis | UMLS:ICD10CM:B37.3 | Candidiasis of vulva and vagina |
|  | | Diagnosis | UMLS:ICD10CM:B37.49 | Other urogenital candidiasis |
|  | **Settings for the performed analyses** | | | |
|  | | Risk analysis | | including patients with outcome prior to the time window |
|  | | Kaplan - Meier survival analysis | | including patients with outcome prior to the time window |
| Dehydration/Volume depletion | | | | |
|  | **Outcome definition** | | | |
|  | | Diagnosis | UMLS:ICD10CM:E86.0 | Dehydration |
|  | | Diagnosis | UMLS:ICD10CM:E86.9 | Volume depletion, unspecified |
|  | **Settings for the performed analyses** | | | |
|  | | Risk analysis | | including patients with outcome prior to the time window |
|  | | Kaplan - Meier survival analysis | | including patients with outcome prior to the time window |
| Palpitation | | | | |
|  | **Outcome definition** | | | |
|  | | Diagnosis | UMLS:ICD10CM:R00 | Abnormalities of heart beat |
|  | **Settings for the performed analyses** | | | |
|  | | Risk analysis | | including patients with outcome prior to the time window |
|  | | Kaplan - Meier survival analysis | | including patients with outcome prior to the time window |
| GB and pancreas disorder | | | | |
|  | **Outcome definition** | | | |
|  | | Diagnosis | UMLS:ICD10CM:K80-K87 | Disorders of gallbladder, biliary tract and pancreas |
|  | **Settings for the performed analyses** | | | |
|  | | Risk analysis | | including patients with outcome prior to the time window |
|  | | Kaplan - Meier survival analysis | | including patients with outcome prior to the time window |
| thyroid cancer | | | | |
|  | **Outcome definition** | | | |
|  | | Diagnosis | UMLS:ICD10CM:C73 | Malignant neoplasm of thyroid gland |
|  | **Settings for the performed analyses** | | | |
|  | | Risk analysis | | including patients with outcome prior to the time window |
|  | | Kaplan - Meier survival analysis | | including patients with outcome prior to the time window |
| CKD stage 5 | | | | |
|  | **Outcome definition** | | | |
|  | | Diagnosis | UMLS:ICD10CM:N18.5 | Chronic kidney disease, stage 5 |
|  | **Settings for the performed analyses** | | | |
|  | | Risk analysis | | including patients with outcome prior to the time window |
|  | | Kaplan - Meier survival analysis | | including patients with outcome prior to the time window |
| Dialysis | | | | |
|  | **Outcome definition** | | | |
|  | | Diagnosis | UMLS:ICD10CM:N18.6 | End stage renal disease |
|  | | Diagnosis | UMLS:ICD10CM:Z99.2 | Dependence on renal dialysis |
|  | | Procedure | UMLS:CPT:1012740 | Dialysis Services and Procedures |
|  | | Procedure | UMLS:CPT:1029674 | Dialysis Circuit Procedures |
|  | **Settings for the performed analyses** | | | |
|  | | Kaplan - Meier survival analysis | | including patients with outcome prior to the time window |
|  | | Risk analysis | | including patients with outcome prior to the time window |
| Renal transplant | | | | |
|  | **Outcome definition** | | | |
|  | | Procedure | UMLS:CPT:1008098 | Renal Transplantation Procedures |
|  | **Settings for the performed analyses** | | | |
|  | | Risk analysis | | including patients with outcome prior to the time window |
|  | | Kaplan - Meier survival analysis | | including patients with outcome prior to the time window |
| AMI | | | | |
|  | **Outcome definition** | | | |
|  | | Diagnosis | UMLS:ICD10CM:I21 | Acute myocardial infarction |
|  | **Settings for the performed analyses** | | | |
|  | | Risk analysis | | including patients with outcome prior to the time window |
|  | | Kaplan - Meier survival analysis | | including patients with outcome prior to the time window |
| Stroke | | | | |
|  | **Outcome definition** | | | |
|  | | Diagnosis | UMLS:ICD10CM:I61 | Nontraumatic intracerebral hemorrhage |
|  | | Diagnosis | UMLS:ICD10CM:I62 | Other and unspecified nontraumatic intracranial hemorrhage |
|  | | Diagnosis | UMLS:ICD10CM:I63 | Cerebral infarction |
|  | **Settings for the performed analyses** | | | |
|  | | Kaplan - Meier survival analysis | | including patients with outcome prior to the time window |
|  | | Risk analysis | | including patients with outcome prior to the time window |
| cardiac arrest | | | | |
|  | **Outcome definition** | | | |
|  | | Diagnosis | UMLS:ICD10CM:I46.2 | Cardiac arrest due to underlying cardiac condition |
|  | | Diagnosis | UMLS:ICD10CM:I46.9 | Cardiac arrest, cause unspecified |
|  | **Settings for the performed analyses** | | | |
|  | | Kaplan - Meier survival analysis | | including patients with outcome prior to the time window |
|  | | Risk analysis | | including patients with outcome prior to the time window |

**Supplementary Table 6**. Clinical outcomes comparing patients on combination therapy (GLP-1RA and SGLT2i) versus on monotherapy (SGLT2i)

|  | **GLP-1RA + SGLT2i (16,224)** | **SGLT2i (16,224)** | **Risk difference (95% CI)** | **Relative Risk Reduction* (%)** | **Hazard ratio (95% CI)** | **P-value** | **E-value (E-value for value the upper limit of CI)** |
| --- | --- | --- | --- | --- | --- | --- | --- |
| **Primary Outcome** |  |  |  |  |  |  |  |
| MAKE | 1,590 (9.80) | 2,158 (13.30) | -0.04 (–0.04, –0.03) | 28.51 | 0.70 (0.65, 0.74) | < 0.001 | 1.89 (0.744) |
| CKD Stage 5 | 318 (1.96) | 395 (2.43) | -0.01 (–0.01, –0.00) | 23,13 | 0.77 (0.66, 0.89) | < 0.001 | 1.70 (1.39) |
| Dialysis/ ESKD | 906 (5.58) | 1,038 (6.40) | -0.01 (–0.01, –0.00) | 16.16 | 0.83 (0.76, 0.91) | < 0.001 | 1.53 (1.33) |
| Renal Transplant | 10 (0.06) | 10 (0.06) | 0.00 (–0.00, 0.00) | -50.00 | 1.44 (0.51, 4.04) | 0.487 | 1.89 (4.60) |
| **Secondary Outcomes** |  |  |  |  |  |  |  |
| All-Cause Mortality | 586 (3.61) | 1,004 (6.19) | -0.03 (–0.03, –0.02) | 43.52 | 0.55 (0.50, 0.61) | < 0.001 | 2.38 (2.15) |
| MACE | 2,419 (14.91) | 2,805 (17.29) | -0.02 (–0.03, –0.02) | 16.04 | 0.83 (0.78, 0.87) | < 0.001 | 1.55 (1.43) |
| AMI | 1,141 (7.03) | 1,155 (7.12) | -0.00 (–0.01, 0.01) | 4.45 | 0.95 (0.88, 1.03) | 0.221 | 1.23 (1.17) |
| Stroke | 1,032 (6.36) | 1,075 (6.63) | -0.00 (–0.01, 0.00) | 7.30 | 0.92 (0.85, 1.01) | 0.070 | 1.30 (1.07) |
| Cardiac Arrest | 142 (0.88) | 236 (1.45) | -0.01 (–0.01, –0.00) | 42.41 | 0.58 (0.47, 0.71) | < 0.001 | 2.29 (1.85) |

**Supplementary Figure 1.** Subgroup analysis of hazard ratios for MAKE.

**Supplementary Figure 2.** Subgroup analysis of hazard ratios for all-cause mortality.

**Supplementary Figure 3.** Subgroup analysis of hazard ratios for MACE.
